# Cardiac myxoma with high standardized uptake value of FDG-PET-CT in the right ventricular outflow tract

**DOI:** 10.1186/s44215-024-00179-x

**Published:** 2024-12-20

**Authors:** Hiroo Uehara, Kenta Ohba, Makoto Ono, Tomohiro Imazuru, Tomoki Shimokawa

**Affiliations:** Department of Cardiovascular Surgery, Teikyo University Hospital, 2-21-1 Kaga, Itabashi-Ku, Tokyo, 173-8606 Japan

**Keywords:** Cardiac myxoma, Right ventricle, Positive FDG PET/CT uptake

## Abstract

**Background:**

Cardiac myxoma rarely occurs in the right ventricle, and as is a benign disease, it rarely shows high positivity on 18F fluorodeoxyglucose (FDG) positron emission tomography/computed tomography (PET/CT).

Case presentation.

We present herein the case of a 77-year-old woman who was found to have a heart murmur during a routine health checkup. Further examination revealed a 27-mm tumor in the right ventricular outflow tract (RVOT) and moderate aortic valve stenosis. Additionally, during her preoperative evaluation, she was diagnosed with a 10-mm tumor in the right breast, prompting her referral to our hospital for further evaluation and treatment. Contrast-enhanced CT and magnetic resonance imaging of the chest did not show signs strongly suggestive of malignancy, nor did echocardiography. However, FDG-PET/CT showed an abnormally high standardized uptake value (SUV) max of 9.91. Based on these findings, we decided the best treatment course was tumor resection of the RVOT and aortic valve replacement. Our intraoperative examination confirmed a tumor inferior to the pulmonary valve; therefore, we resected three branches of the septal artery feeding the tumor, including part of the right ventricular free wall. A rapid pathological examination indicated a benign tumor, and the patient’s final diagnosis was a cardiac myxoma. The postoperative course was uneventful, and to date, workup including CT scans during follow-up has shown no obvious recurrence.

**Conclusion:**

This case highlights the challenges and importance of accurate imaging diagnoses in cardiac tumors. The patient underwent a successful surgical resection of the cardiac myxoma, emphasizing the need for careful postoperative follow-up.

## Background

Among autopsy cases, the incidence of cardiac tumors is reported to be 0.0017–0.33% [1], with primary cardiac tumors accounting for 24–37% of these tumors. Furthermore, most of the primary cardiac tumors are cardiac myxomas [2], which are typically benign and do not show uptake on 18F fluorodeoxyglucose (FDG) positron emission tomography/computed tomography (PET/CT). However, we report herein a case of cardiac myxoma with an abnormally high standardized uptake value (SUV) max (9.91) on FDG-PET/CT.

## Case presentation

**Fig. 1:**
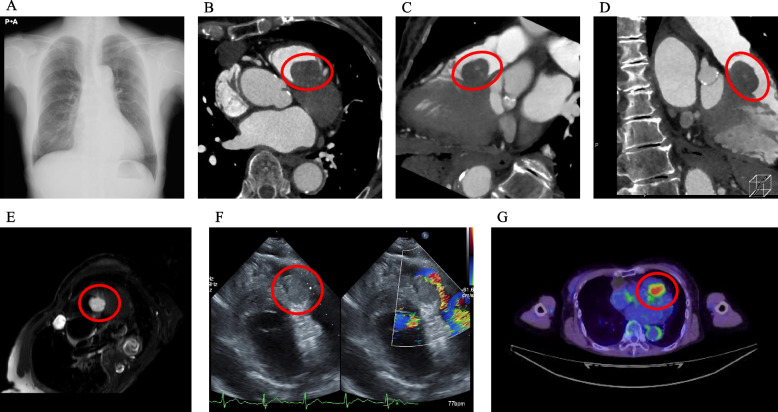
Preoperative findings. **A** Chest X-ray. **B**, **C**, **D** Computed tomography. **E** Magnetic resonance imaging (T2W). **F** Transthoracic echocardiography. **G** FDG-PET/CT (a tumor shown as a red circle)

The patient was a 77-year-old woman. She was diagnosed with a heart murmur during a routine health checkup, after which transthoracic echocardiography revealed moderate aortic valve stenosis and a 27-mm cardiac mass in the right ventricular outflow tract (RVOT). Additionally, during her preoperative evaluation, she was diagnosed with a 10-mm mass in her right breast, resulting in her referral to our hospital for further assessment and treatment. No past medical history or family history, non-smoker, occasional alcohol consumption. She was of average intelligence, and her height, weight, and body surface area were 153.1 cm, 46.0 kg, and 1.403 m^2^, respectively. She had a systolic murmur in the second right intercostal space, otherwise unremarkable physical findings. Her blood test on admission showed almost all chemical parameters were within the normal range, but a cardiac marker (N-terminal prohormone of brain natriuretic peptide: 258.0 pg/mL) and some tumor markers (cancer antigen 15–3: 6.2 U/mL, neuron-specific enolase: 30.2 ng/mL) were high. Chest X-ray showed no significant findings with 45.6% of cardiothoracic ratio (Fig. 1A). Contrast-enhanced CT showed well-defined 22.9 × 18.1 × 21.7 mm soft tissue mass in the RVOT with heterogeneous enhancement and 10 mm enhancing solid tumor in the right breast (Fig. 1B, C, D).

Additionally, magnetic resonance imaging (MRI) revealed a high-signal mass in the RVOT on T2-weighted images with enhancement after gadolinium contrast (Fig. 1E) and 13.0 × 7.2 × 21.0 mm mass in the right breast. Transthoracic echocardiography showed moderate aortic valve stenosis (peak pressure gradient of 39 mmHg, mean pressure gradient of 20 mmHg, peak flow velocity of 313 cm/s, and effective orifice area of 1.42 cm^2^) and 30 × 14 mm tumor in the RVOT with clear boundaries and no significant blood flow signal (Fig. 1F). FDG-PET/CT demonstrated 30-mm tumor in the RVOT with an SUV max of 9.91 (Fig. 1G) and 10-mm tumor in the right breast with mild-to-moderate accumulation. Based on the echocardiography, contrast-enhanced CT, and MRI, the tumor in the RVOT was not suggestive of malignancy. However, FDG-PET/CT showed abnormal uptake in the RVOT. Differential diagnoses included benign (cardiac myxoma) and malignant (cardiac myxosarcoma or cardiac breast cancer metastases) options. Considering the patient’s age and frailty, we discussed the case in a heart team conference and adopted a policy to perform the aortic valve replacement and diagnosis of the tumor.

**Fig. 2:**
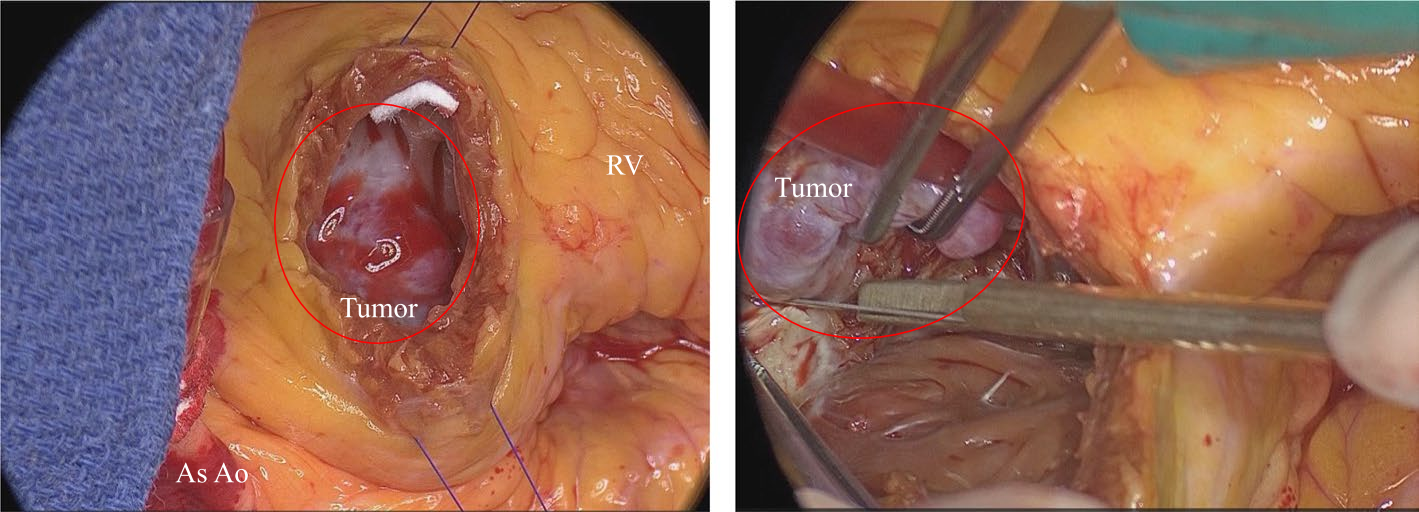
Intraoperative findings. As Ao, ascend aorta; RV, right ventricle

**Fig. 3:**
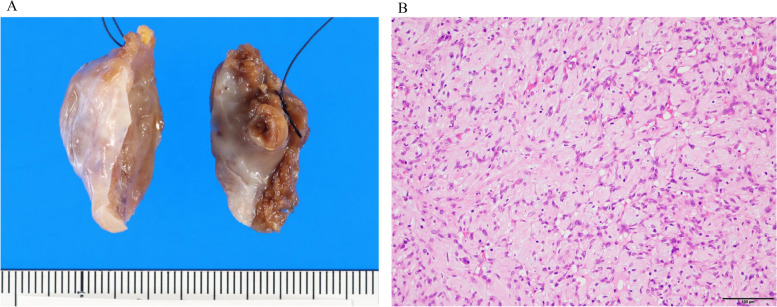
Pathological assessmssent

A median sternotomy was performed under general anesthesia, which allowed us to open the pericardium and visualize the tumor inferior to the pulmonary valve. A 5-cm vertical incision was done in the right ventricular free wall, parallel to the left anterior descending artery. The tumor with an unclear border with the myocardium was exposed (Fig. 2). Subsequently, we resected nutrient vessels from three branches of the left anterior descending artery and the tumor along with part of the right ventricular free wall. Intraoperative frozen section diagnosis showed cardiac myxoma with negative margins. After the right ventricular incision was closed in two layers with felted 4–0 monofilament sutures, the aortic valve replacement was performed. Total operative time was 270 min with a cardiopulmonary bypass time of 164 min and aortic cross-clamp time of 113 min. The postoperative recovery was uneventful, following extubation on the first day after surgery. Postoperative echocardiography and contrast-enhanced CT showed no residual tumor, and she was discharged home on postoperative day 25. Macropathological findings revealed the tumor of 33 × 20 × 15 mm with pale brown and gelatinous (Fig. 3A), and histological examination revealed the tumor composed of spindle-shaped-to-ovoid cells in a myxoid stroma with collagen fibers and inflammatory cell clusters with lymphocytes and plasma cells (Fig. 3B). The tumor cells were positive for CD34 but negative for AE1/AE3, S-100, and calretinin. The myxoid stroma was positive for Alcian blue and periodic acid–Schiff (PAS) staining. The patient’s final diagnosis, therefore, was cardiac myxoma.

## Discussion

Preoperative evaluations often do not yield a definitive classification for cardiac tumors, as in the present case. Approximately, 70% of surgically resected cardiac tumors are benign [1, 3] with an incidence of all cardiac tumors 0.0017–0.33% reported among autopsy cases [1]. Myxomas are the most frequent primary cardiac tumor (24–37%), followed by angiosarcomas (7.3–8.5%) and papillary fibroelastomas (7.9–8.0%) [2]. Metastatic cardiac tumors are found among 10–20% of autopsy cases, while 1.7–9.0% of autopsy cases reveal malignant tumors, which are much more common than primary cardiac tumors [3]. The most common malignancy that metastasizes to the heart is lung cancer (33%), followed by breast cancer, malignant melanoma, leukemia, and malignant lymphoma.

One of the preoperative differential diagnoses in this case was cardiac myxoma, which are more common in women (59.4%) and largely occur in the 6th and 7th decades of life. They typically originate from the atrial septum, with the left atrium being the most common site of origin (84.2–88.3%), followed by the right atrium (9.4–10.5%) and right ventricle (1.2–5.3%) [4]. Multiple tumors may be suggestive of Carney syndrome, which involves skin and mucosal pigmentation as well as adrenal cortical hyperplasia.

Cardiac myxomas may present with three classic symptoms: intracardiac obstruction, constitutional signs, and embolic phenomena [5]. However, many are asymptomatic and incidentally discovered during routing examinations due to advancements in transthoracic echocardiography, which is the most useful diagnostic tool in these cases, allowing for the assessment of the size, shape, attachment, stalk, mobility, and location of the tumor, as well as its impact on intracardiac hemodynamics. CT is useful for evaluating the relationship of the tumor with other organs and vessels, as well as aiding surgical planning. MRI shows uniform isointense signals on T1-weighted images and hyperintense signals on T2-weighted images, which helps differentiate it from other cardiac tumors [6].

FDG PET/CT uptake patterns in the heart are categorized as follows: none, diffuse, focal, and focal on diffuse. Non-tumorous conditions, such as atrial septal fatty hypertrophy, ischemic coronary artery disease, Takotsubo cardiomyopathy, hypertrophic cardiomyopathy, and cardiac sarcoidosis, can show focal uptake [7]. In cardiac tumors, however, FDG PET/CT is useful for distinguishing between benign and malignant lesions. Some previous studies demonstrated that benign, primary malignant, and secondary malignant cardiac tumors typically showed SUV max of 2.8 ± 0.6, 8.0 ± 2.1, and 10.8 ± 4.9, respectively, with cutoff values of SUV max 3.5 (sensitivity 100% and specificity 86%) to 4.6 (sensitivity 94% and specificity 100%) [8, 9]. Additionally, the SUV max of cardiac myxoma was reported to be moderate, ranging from 1.2 to 5.3 [10, 11].

In the present case, the SUV max of the cardiac myxoma was significantly higher than expected (9.91), although no other preoperative findings suggested malignancy. The high SUV max in this case, therefore, warranted surgical resection for diagnostic and therapeutic purposes. The reason for the high SUV max is unclear from the pathological and macroscopic findings.

Surgical resection is the primary treatment for cardiac myxomas, preferably as soon as possible after the tumor is diagnosed to mitigate the risk of embolism and cardiac obstruction, which can result in preoperative mortality [12]. Additionally, complete resection with clear margins is crucial as recurrence is a concern from the resection margins. The postoperative prognosis is generally favorable, with a recurrence rate of 1–4% for spontaneous cases and 22% for familial or complex cases, with recurrence occurring more frequently in younger patients.

In the present case, the pathological diagnosis was cardiac myxoma. In light of the patient’s age, the short- and long-term prognoses after surgical resection were considered to be relatively good. However, there have been case reports of post-resection brain metastases, as well as other malignant tumors due to postoperative recurrence [13, 14]. As the present case showed high accumulation on FDG PET/CT, careful postoperative follow-up was necessary.

## Conclusion

The present case highlights the challenges and importance of accurate imaging diagnosis in cardiac tumors. The patient underwent the successful surgical resection of a cardiac myxoma with a high SUV max on FDG-PET/CT, emphasizing the need for careful postoperative follow-up.

## Data Availability

The datasets used are available from the corresponding author on reasonable request.

## Abbreviations

FDG: 18F fluorodeoxyglucose
MRI: Magnetic resonance imaging
PET/CT: Positron emission tomography/computed tomography
RVOT: Right ventricular outflow tract
SUV: Standardized uptake value
